# Development of Filter Media by Electrospinning for Air Filtration of Nanoparticles from PET Bottles

**DOI:** 10.3390/membranes11040293

**Published:** 2021-04-19

**Authors:** Daniela P. F. Bonfim, Fabiana G. S. Cruz, Vádila G. Guerra, Mônica L. Aguiar

**Affiliations:** Departamento de Engenharia Química, Federal University of São Carlos, Washington Luis Road, km 235, São Carlos, SP 13565-905, Brazil; bonfimdaniela22@gmail.com (D.P.F.B.); fabigscruz@gmail.com (F.G.S.C.); vadila@ufscar.br (V.G.G.)

**Keywords:** filter media, electrospinning, air filtration, nanotechnology, quality factor, recycling materials

## Abstract

Air pollution and solid pollution are considered global problems, and endanger human health mainly due to the emission of fine particulate matter released into the atmosphere and improper disposal of post-consumer plastic bottles. Therefore, it is urgent to develop filter media to effectively protect the public. The properties of plastics make them potential candidates for nanofiber mat formers due to their attractive structural and mechanical characteristics. This work aims to produce and evaluate novel PET electrospun fibers dispensed with the use of support materials to be used as filter media to remove nanoparticles from the air. The electrospinning process was carried out by changing the concentration of the polymer solution, the needle diameter, and the electrospinning processing time at two rotation speeds. The average diameters of the micro- and nanofibers of the filter media produced ranged from 3.25 μm to 0.65 μm and it was possible to conclude that, as the size of the fibers decreased, the mechanical strength increased from 3.2 to 4.5 MPa. In filtration tests, a collection efficiency of up to 99% with low-pressure drops (19.4 Pa) was obtained for nanoparticles, demonstrating high quality factor filter media, which could be applicable in gas filtration.

## 1. Introduction

In recent decades, air pollution has endangered human health, especially due to the emission of fine particulate matter into the atmosphere by industrial processes and auto-vehicles, and exposure to outdoor particles has been strongly associated with adverse health effects [1,2,3]. Particularly, fine particles with a diameter less than 2.5 μm (PM2.5) in a complex mixture have been acknowledged as the main hazard because they can easily penetrate deep into human lungs and bronchi [4,5]. This persistent and worldwide air pollution problem endangers public health due to the existence of poisonous pollutants, which are a mixture of particles, toxic gases, and microorganisms [6]. Another major problem that causes great environmental concern is urban solid waste pollution, like polyethylene terephthalate (PET) packaging, as it is inadequately disposed of after consumption [7,8]. Considering this damage, it is necessary to investigate new technological processes of recycling or transformation for this polymeric residue.

A filtration process using fibrous filter media is widely applied in the removal of particulate matter from air streams due to their simplicity of use, low cost, and ability to achieve high collection efficiencies [9,10,11,12]. However, more studies are necessary regarding the filtration of nanoparticles. Currently, nanofiber membranes have presented superior performance compared to traditional microfiber filtration materials [9,13,14]. For this reason, nanofibers have become the major interest of research in various industrial fields, including sensors, tissue engineering, fuel cells, capacitors, and filtration [15,16,17,18]. In addition, they stand out because of their desirable properties for filtration, such as mechanical strength, elasticity, porosity, and charged surface area, among others [17].

The advancement and rapid dissemination of nanoscience and technology concepts have enabled the development of nanomaterials, the most prominent of which is nanofibers, obtained through a production process called electrospinning. This method uses electrostatic force to obtain fibers with high surface area compared with those produced by other methods. It is important to highlight that this method is distinguished from others by the ability to control the diameter, morphology, orientation, and fiber structure [17]. However, one of the challenges in producing these filter media is the adjustment and control of several parameters which highly influence fiber production, such as polymer concentration, solvent ratio, collection time (or electrospinning processing time), tip to collector distance, needle diameter, and the applied voltage [14,17,19,20,21]. A wide variety of polymers have been used in the preparation of these nanofibers, e.g., polyamide, polyimide, polystyrene, polyacrylonitrile, polyacid acetic, and polyvinyl acetate. When applied to air filtration, those nanofibers reached efficiency values close to 98.79% and 96.79% for nanoparticles (100 and 300 nm in diameter) [14,22]. The use of polyethylene terephthalate has also been reported and the filter media achieved high performance for the filtration of hydrocarbons and particulate matter of cigarette smoke. Efficiency values close to 93.7% were reached for the remediation of hexavalent chromium in contaminated waters [16,23].

It is important to emphasize that the study of recycled materials has awakened great interest in recent years due to the need to reduce waste and the search for new sources of materials. The properties of polyester polymers also make them potential candidates for nanofiber mat formers due to their attractive structural and mechanical characteristics [24]. Currently, in the civil construction industry, post-consumer PET has been used in the production of nanocomposites and research has already been done about their use in the development of filter media [11,16,25,26]. However, current works refer to liquid filtration using recycled PET.

Therefore, the objective of this work was to produce microfiber and nanofiber materials for use in nanoparticle filtration operations from polymer solutions of post-consumer PET bottles at different concentrations. To achieve that goal, some parameters of the electrospinning process were varied—polymer concentration, needle diameter, rotation speed, and electrospinning processing time—and their influence on fiber morphology and filtration performance was analyzed. The morphology of PET fibers was examined by scanning electron microscopy (SEM), thickness, mechanical properties, permeability, and pressure drop. In addition, filtration performances were determined by measuring the penetration of sodium chloride (NaCl) aerosol particles (9 to 300 nm in diameter) using scanning mobility particle sizers (SMPS). The main objective was to obtain sustainable filter media with high mechanical resistance that dispenses with the use of microporous substrate and that demonstrate a better PM removal effect, characterized by high values of the quality factor (i.e., high filtration efficiency and low-pressure drop).

## 2. Materials and Methods

### 2.1. Materials

Trifluoroacetic acid (TFA—Neon), dichloromethane (DCM—Synth), 10 mL plastic syringes (BDPlastipak, Curitiba, Brazil), and needles with diameters of 0.7 mm and 0.3 mm (BDPrecisionGlide, Curitiba, Brazil) was used. Sodium chloride (NaCl—Sigma-Aldrich (St. Louis, MO, USA), 99%) was used to generate nanoparticles to evaluate the removal efficiency.

### 2.2. Electrospinning process

The fibers were prepared by electrospinning using a PET polymer solution (20, 12, and 10 wt%) dissolved in TFA and DCM (70/30 wt%). All the bottles in the experiment were reused from 500 mL clear soda packaging, of which only the central part of the bottles was used. The bottles were cleaned and rinsed with pure ethanol and dried, followed by shredding into small pieces. All solutions were maintained under magnetic stirring at room temperature for 3 h and then the PET solution was loaded into a 12 mL syringe, with an attached needle of different diameters (0.3, 0.55, and 0.7 mm). In all experiments, a voltage of 20 kV (High Voltage Power Supply, T1CP 300 304n-iSeg), a flow rate of 0.8 mL/h (syringe pump, KDS 100, KDScientific, Holliston, MA, USA), collector speed at 357 and 177 rpm, and a needle–collector distance fixed at 10 cm were employed. The electrospinning process was carried out for 3 and 6 h. Energy-dispersive X-ray spectroscopy analysis (EDX, Bruker XFlash 6/60, Germany) to measure the atomic percentage in PET bottles and virgin PET. Viscosity was measured using a Brookfield spindle SC4-18 viscometer (Brookfield LV-DVIII, Brookfield Engineering Laboratories Inc., Middleboro, MA, USA). The filter media were named PET20%, PET12%, and PET10% according to the concentration of the polymer. For the polymer concentration of 10%, the needle diameter was also varied, and the samples were named PET10%_0.3 and PET10%_0.55. Table 1 shows the main operational parameters.

### 2.3. Characterization of the electrospun mats

Scanning electron microscopy (SEM, Philips XL30FEG, Netherlands) was employed to characterize the morphology of the fibers (size diameter distribution and thickness) by image analysis software (Image J1.29X), according to the method described by Salussoglia, Tanabe, and Aguiar (2020) [27]. The samples were platinum sputter-coated before the SEM analysis. The mechanical properties of the membranes were tested on a TA (Model Q800) whose sample size was 5.40 to 5.50 mm in length by 6.80 to 7.00 mm in width. Material elongation was performed at a speed of 700 µm/min^−1^ from 0 to 10,000 µm at room temperature (25 °C).

Filter media permeability experiments were performed, varying the flow rate from 100 to 2000 mL·min^−1^. The permeability constant (K_1_) was determined using Equation (1):

∆P/L = (μ/K_1_) × (V_s_) (1)

where L represents the thickness of the filter, µ is the viscosity of the fluid (air), K_1_ is the permeability constants of the filter, and (V_s_) is the surface velocity. The pressure drop (∆P) was measured using a digital manometer (VelociCalc Model 3A-181WP09, TSI, Shoreview, MN, USA) connected to the filtration apparatus. The methodology employed here was as described in [28].

The porosity of the fibrous membranes can be calculated by Ergun’s equation (1952), as follows (Equation (2)):

∆P/L = (150(1 − ε)^2^μv_s_)/(ε^3^ d_f_^2^) + (1.75(1 − ε) ρ_g_ v_s_^2^)/(ε^3^d_f_) (2)

where ρ_g_ is the gas density and d_f_ is the average diameter of the fiber.

### 2.4. Evaluation of PM removal efficiencies

Filtration tests were performed, keeping the surface velocity (4.8 cm s^−1^), the flow rate (1500 mL min^−1^), and the filtration area (5.2 cm^2^) constant. It was possible to obtain the particle diameter distribution at the beginning of filtration from a 0.1 g L^−1^ solution of NaCl in which the nanoparticles generated had a diameter ranging from 7.37 to 150 nm (0.007 to 0.15 µm). The collection efficiency was experimentally obtained through the technique of electric mobility, in which the number of particles was calculated before and after the passing of air through the filter media, determined by Equation (3):

E = (C_0_ − C_1_)/C_0_ (3)

where C_0_ and C_1_ represent the concentration of nanoparticles before and after the air passed through the filter media, respectively. The quality factor (Q_f_) is used to evaluate the overall performance of the filter media and can be determined as follows (Equation (4)):

Q_f_ = (− ln(1 − E))/∆P (4)

The experimental unit used in this paper consisted of an air compressor (Shultz Acworth, GA, USA), air purification filters (Model A917A-8104N-000 and 0A0-000), atomizer aerosol generator (Model 3079, TSI, Shoreview, MN, USA), diffusion dryer (Norgren IMI, Birmingham, UK), krypton and americium neutralizing source (Model 3054, TSI, Shoreview, MN, USA), filter apparatus, flow meter size 3 (Gilmont, Vernon Hills, IL, USA), and SMPS device formed by an electrostatic classifier (Model 3080, TSI, Shoreview, MN, USA), differential mobility analyzer, and ultrafine particle counter (Model 3776, TSI, Shoreview, MN, USA), as described by Bortolassi et al. (2019) [29].

## 3. Results

The tests were performed in order to verify the effect of the variation of solution concentration, needle diameter, collector speed, and electrospinning processing time on the morphological properties of the fibers, while other parameters were maintained constant according to common conditions reported in the literature [14,16,23,26] for fiber production by electrospinning. The filter media were characterized by scanning electron microscopy (SEM). Thickness, porosity, permeability, and pressure drop were also determined. In addition, filtration performance tests were evaluated.

### 3.1. Morphological characteristics of the fibers

**Figure 1:**
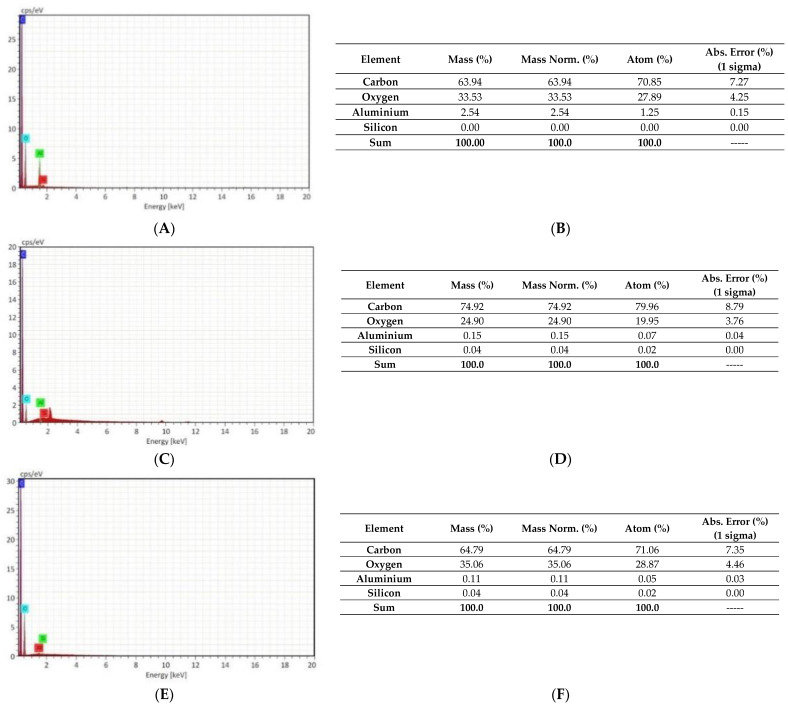
Energy dispersive X-ray spectroscopy (EDX) mapping of the elements carbon, oxygen, silicon, and aluminum: (**A**) Energy dispersive X-ray spectroscopy of virgin PET, (**B**) elemental analysis of virgin PET, (**C**) energy dispersive X-ray spectroscopy of PET bottle, (**D**) elemental analysis of PET bottle, (**E**) energy dispersive X-ray spectroscopy of PET20% and (**F**) elemental analysis of PET20%.

The EDX analysis made it possible to compare the composition of the materials used [29,30]. Figure 1 shows the energy dispersive X-ray spectroscopy (EDX) and elemental analysis of fibers and PET in the form of bottles and pure polymer.

It can be concluded that the main elements found in the two materials, carbon and oxygen, appear in similar quantities, with carbon with a mass percentage of 64% for virgin PET and 74% for PET bottles and oxygen at 34% and 25%, respectively, and the remaining elements (silicon and aluminum) in much smaller percentages. Based on the atomic mass composition ratios (in percentages) of virgin PET to bottle PET, it was possible to observe that the relationships between them are within the error range, that is, the compositions have not undergone major changes. Existing variations may possibly be due to the bottle manufacturing process and the wear and tear of the polymer after bottle production [31]. In this way, it can be inferred that there was no significant change in the composition that compromised the use of plastic bottles as a polymeric source for the production of fibers. It is also possible to observe that the main elements found in the fibers, carbon and oxygen, appear in similar quantities to the virgin PET, 65% of carbon and 35% of oxygen, which also allows us to conclude that the relationships between the precursors and the products are within the error range, that is, the compositions have not undergone major changes. 

Electrospun mats were successfully prepared in different conditions from recycled PET solutions and the morphological characteristics that will be discussed in this session are presented in Table 2.

The first tests were done with polymeric solutions whose concentration was equal to 10% wt%. As can be seen in Table 2, the reduction in the diameter of the needle led to a small reduction in the average fiber diameter, as is exemplified by the samples PET10%_0.55 and PET10% _0.3 (equal experimental conditions, Table 1). It is also possible to verify that, although the rotation speed is different, this variation does not significantly change the average fiber diameter, as can also be observed when comparing the PET10% and PET10%_0.3 samples. By reducing the speed of rotation from 357 to 177 rpm, the average fiber diameter showed a small reduction. As reported in the literature, polymer concentration is more significant in the distribution of diameters [14].

**Figure 2:**
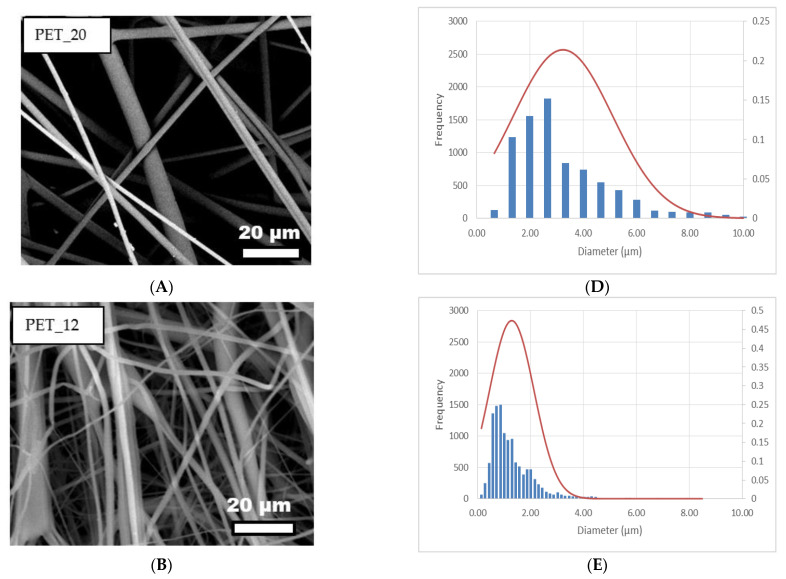

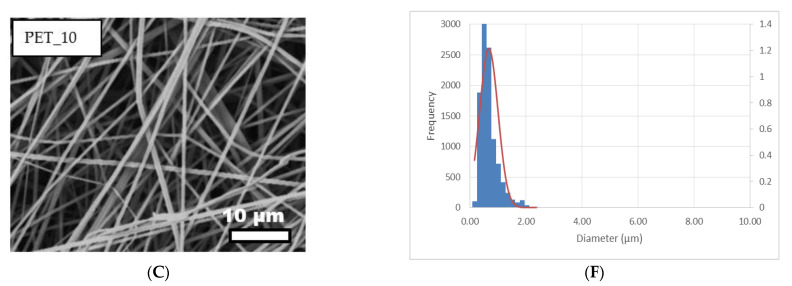
SEM images of electrospun fibers: PET20% (**A**), PET12% (**B**), and PET_10% (**C**), with their respective fiber size distributions (**D**–**F**).

As the samples with a concentration equal to 10% did not show larger variations in diameter, it was decided to base the main discussion on PET10%. The reduction of the polymeric concentration from 20 to 10% wt% also resulted in a reduction in the average fiber diameter and, as can be seen, the reduction was from 3.25 to 1.29 µm. Fiber diameters were measured from SEM images using image analysis software (Image J1.29X) as described in the literature [27]. Figure 2 shows scanning electron microscopy (SEM) fiber images (PET20%, PET12%, and PET10%) and their corresponding size distributions that were produced in order to investigate the morphological features of fibers after electrospinning. The bar in Figure 2 shows the measurement distribution, whereas the line is an approximation of the distribution function based on a Gaussian distribution.

After obtaining the fibers, it was possible to analyze the influence of the processing parameters on the fiber morphology, and it was observed that the reduction in solution concentration led to a decrease in the mean diameter.

This result is in agreement with literature data showing that thinner fibers are obtained from solutions with lower concentrations [14,19,21]. The concentration of the solution was the parameter that most interfered in the distribution of average diameters, since concentration is closely associated with viscosity, which highly influences the formation of the drop solution and consequently modifies the electrospinning jet [18,21]. The viscosities of the solutions were measured and presented values of 1286.7, 221.7, and 172.3 cP for PET20%, PET12%, and PET_10%, respectively. Solutions with very low polymer concentrations result in low viscosities, which cannot resist fiber deformation over the applied electric field before reaching the collector. However, very high concentrations result in high viscoelastic forces, which resist elongation during the process, resulting in larger fiber diameters [32]. Consequently, viscosity is one of the most significant parameters that influences fiber diameter, and the higher the viscosity, the larger the fiber diameter, as shown in Figure 1D–F. In this paper, it is possible to verify the effect of the polymeric concentration on the average diameter when comparing the PET20% and PET12% samples, since the other parameters remain constant. The reduction in the polymeric concentration from 20 to 12% wt% resulted in a decrease in the average fiber diameter from 3.25 to 1.29 µm (Figure 2).

Needle diameter was also an investigated variable, that varied from 0.7 mm to 0.3 mm, as shown in Table 2. Katti and Robinson (2004) presented a study analyzing the influence of needle diameter on fibers resulting from electrospinning and concluded that reducing the needle diameter resulted in thinner fibers [33]. This result was also confirmed by other researchers [21,34]. The variation in needle diameter is directly proportional to fiber diameter. For larger diameter needles, the diameter of the formed fibers is also larger due to the larger amount of solution available on the tip of needle, contributing to the formation of a larger Taylor cone, a larger initial jet, and, hence, a larger diameter fiber [35]. In smaller diameter needles, the size of the drop formed at the needle tip is also smaller, increasing the surface tension of the drop and consequently increasing the electrostatic force required to initiate the solution jet. Thus, jet acceleration decreases and the fiber has more time to stretch before being deposited in the collector, resulting in thinner fibers with smaller diameters. However, the reduction in needle diameter has a limit, as very small diameters may prevent jet formation from the droplet in the needle orifice [34]. This behavior was verified in this study, since the reduction of the needle diameter from 0.55 to 0.3 resulted in thinner fibers, respectively, for the PET10%_0.55 and PET10%_0.3 samples (Table 2). Therefore, it is possible to infer that the combined effect of these two variables (polymer concentration and needle diameter) led to a reduction in the fiber diameter of the PET20%, PET12%, and PET10% samples from 3.25 to 1.29 to 0.67 µm.

Regarding the collection time of fibers, Matuvalecius and contributors (2014) studied different conditions, with results showing that fiber thickness and weight depend on the distance from the needle to the collector, as well as on the collection time [36]. As in this study the distance between needle and collector was kept constant, the thickness variations were a result of the reduction in collection time from 6 h to 3 h. For samples PET20% and PET12%, which had a collection time of 6 h, the thickness of the fibers produced were close (the difference resulting from the reduction in the average diameter), while for sample PET10%, the thickness was smaller because the electrospinning processing time was smaller, as can be seen in Table 2. As reported by Guibo et al. (2012), the longer the electrospinning processing time, the greater the thickness of the formed fiber layer (density of fibers under the substrate) and, consequently, the smaller the average pore size [22].

Tensile strength can be determined by how much the material can withstand applied stress. The tensile strength could reach 3.2 MPa, 3.5 MPa, and 4.5 MPa, for samples PET20%, PET12%, and PET10%, respectively (Table 2). As the fiber diameter decreased, an increase in strength was observed. This could be explained by a higher degree of molecular alignment when thinner fibers were produced in the electrical field, due to the increased fiber stretching [24,37]. Thus, the results demonstrate that the fibers exhibited good mechanical properties in the horizontal direction, since no rupture of the media was observed during the tests, ensuring that the fibers have adequate mechanical resistance to the face velocity. These values are close to those reported by other authors for use in filtration operations where fibers were produced by electrospinning PVC with a value of 1.0 MPa [20]. However, Wang et al. (2013) developed a combined PU/PVC membrane, to enhance the mechanical properties, that exhibited excellent filtration performance with a tensile strength close to 9.9 MPa [38].

The investigation of the porosity measurement of various surface layers which are visible in SEM images is very interesting in terms of engineering applications. The porosity of the samples was determined by the Ergun equation [29] and the minimum and maximum pore size by ImageJ software through the analysis of electron microscopy images obtained from each sample as described in the literature [39]. With an appropriate pore size for nanoparticle filtration, formed nonwovens are expected to have desirable filtration efficiency (close to 100%), pressure drop (similar to High efficiency particulate air filter—HEPA filters: 269 Pa), and air filtration capacity [28]. The results for the minimum and maximum pore size and porosity of the samples are also tabulated in Table 3.

The porosity values found were 95.0, 92.4, and 96.7% for samples PET20%, PET12%, and PET10%, respectively, as can be seen in Table 3. Shahrabi and colleagues developed filters (PET/PVP blends) for biomedical applications involving the removal of leukocytes from blood whose porosity was 90.8% as electrospun mats with an average diameter of 1.3 µm [40]. The making of a hybrid PET and aluminum membrane has also been reported by Yun et al. [41], which was reusable for the high-efficiency simultaneous capture and inactivation of airborne microorganisms, and the porosity and average pore size of the PET/Al filter were 75.0% and 119.8 μm, respectively. Additionally, in related literature, PAN electrospun nanofibers whose porosity was similar to that of this work, on the order of 95% for similar ∆P (215.23 Pa) [29], as well as biodegradable nanofibers whose porosity was higher, at 99% [42], are described, and these differences are due to the process of making the filter media.

The interconnected networks of fiber webs significantly improve air filtration efficiency when used as membranes or filters. This is because a smaller fiber diameter usually resulted in a denser network of nanofibers, as shown in Figure 2. With a denser nanofiber network and smaller pore sizes, it would be harder for the particles to pass through. Consequently, smaller fiber diameter corresponded to greater efficiency in the interception and inertial impaction regimes [43].

### 3.2. Permeability, pressure drop and filtration performance of filters media

Permeability is related to the microstructural filter media parameters and indicates the resistance offered to the airflow passage. The collection efficiency measures the filter media’s ability to capture fine particles. This efficiency depends on the particle size to be filtered. The permeability constant, pressure drop, and collection efficiency are summarized in Table 4. The lowest pressure drop value obtained was 13.5 Pa for the PET20% sample.

Sample PET20% presented the highest permeability constant value of 2.2 × 10^−7^. This result may be a consequence of the high concentration used during the PET20% electrospinning. A high concentration of the solution hinders the mobility of polymer chains, as described by Abuzade and contributors (2012) [44]. As can be seen, the sample PET20% presented a higher permeability constant that favors the passage of flow and causes a lower pressure drop during filtration tests, when compared to other samples. The other samples showed permeability values of the same order of magnitude, PET12%, PET10%, PET_10%_0.55, and PET_10%_0.3, and the increase in the permeability constant is due to the smaller diameter of the fibers, as previously discussed [33,34,35]. In addition, the difference in the permeability constant for these samples may be due to the variation in time for electrospinning and rotation speed. Among these samples, PET12% presented a more closed fiber configuration (lower porosity) and, therefore, a lower permeability constant due to higher fiber deposition as a consequence of a longer collection time (6 h). This can be attributed to the fact that an increase in nanofiber layers caused a decrease in void spaces, hindering air flow through the filter and increasing the pressure drop [29]. Wang and co-workers studied the influence of the nanofiber layer on filtration tests and concluded that increasing the number of layers significantly improves filtration efficiency, however, an increase in pressure drop was also observed for a 780 nm particle test diameter [45].

For samples with a polymer concentration of 10% wt%, the differences are related to the rotation speed and the needle diameter. As can be seen, for PET10% and PET_10%_0.3 samples, the higher rotation speed led to a more open structure. The PET10% sample (357 rpm) showed a higher value of the permeability constant, which means less resistance to flow passage. However, as the PET10% sample had greater thickness (220 µm), its pressure drop value (76.1 Pa) was greater than for the PET_10%_0.3 sample, although it had less permeability. For samples with the same rotation speed (PET10%_0.55 and PET10%_0.3), the difference in the permeability constant was due to the smaller fiber diameter, since a smaller fiber diameter usually results in a denser nanofiber network [43].

**Figure 3:**
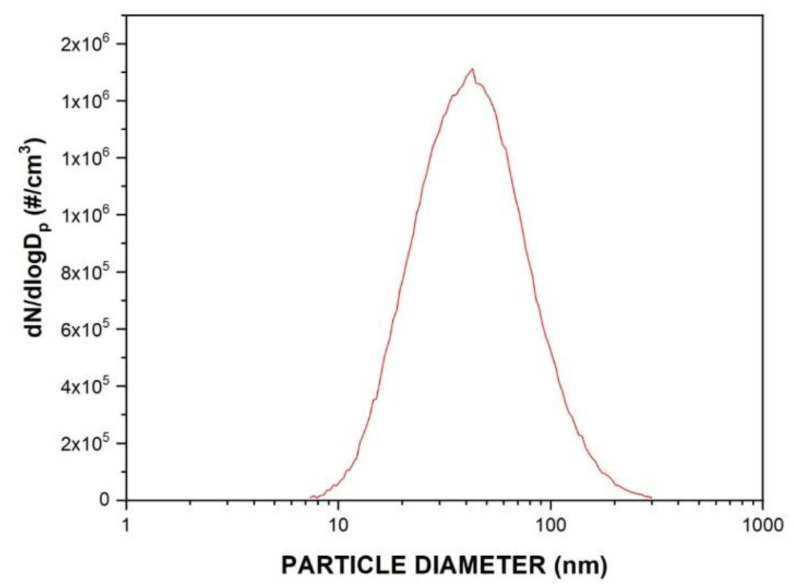
Nanoparticle distribution using NaCl solution.

The particle collection efficiency was also determined for the samples. The efficiency tests were performed with nanoparticles of NaCl with a particle diameter distribution ranging from 7 to 300 nm, as can be seen in Figure 3. Using a particle counter, it was possible to determine nanoparticles distribution generated and, thus, to calculate the global and fractional nanoparticle collection efficiency.

According to the results shown in Table 4, the global collection efficiency increased with the reduction of the average diameter of the fibers. The PET20% sample presented values of 41%, the PET12%, PET10%, and PET_10%_0.55 samples presented a 99% global efficiency, and the PET_10%_0.3 sample presented a 100% global efficiency. The 20% PET sample showed the lowest filtration efficiency (41%), a value already expected, since the literature reports that the efficiency decreases with the increase in the fiber diameter [46]. This is due to the large fiber diameters that hinder the filtration diffusion mechanism in this range of nanoparticles [21,43]. These mechanisms have also been reviewed by Lv et al. (2018) [47] where the efficiency of the filtration of electrospun filters was evaluated. According to Lv et al. (2018), there is a predominance of diffusion and interception mechanisms for particles ranging from 100 to 400 nm.

Woon, Leung, and Sun (2020) [48] also varied the fiber diameter of polyvinylidene fluoride (PVDF) and observed that the reduction of the fiber size led to an increase in the collection efficiency. Almeida et al. (2020) [42] also used NaCl crystals to simulate particulate matter with particle size ranging from 7 to 299 nm and obtained efficiencies of almost 100%. They used fibers of cellulose acetate (CA), which is a biodegradable material, with the cationic surfactant cetyl-piridinyum bromide (CPB).

Leung, Hung, and Yuen (2010) [11] reported that fine nanofiber filters (208 nm) resulted in higher filtration efficiencies for particles 50–200 nm in diameter when compared to thicker nanofibers filters, mainly due to the favoring of the mechanisms of diffusion and interception. This was also observed in this work as, when comparing all samples, the highest global collection efficiency (≈100%) is due to the smaller diameter of the sample fibers [49].

When comparing the characteristics of samples PET12%, PET10%, PET_10%_0.55, and PET_10%_0.3, the global collection efficiency for all is 99%, but this efficiency is a result of different factors in these samples. Sample PET12% has a higher pressure drop, which reflects high collection efficiency, whilst the high collection efficiency of the PET10%, PET10%_0.55, and PET10%_0.3 samples is due to the smaller fiber diameter and uniformity, which favor the nanoparticle capture mechanism. From the results obtained, it can be concluded that the filter media, mainly PET12%, PET10%, PET10%_0.55, and PET10%_0.3, are promising for applications in the mitigation of particulate material, especially PM2.5. Based on this, it can be deduced that for PM2.5, the PET20% filter could achieve a collection efficiency higher than 41%.

**Figure 4:**
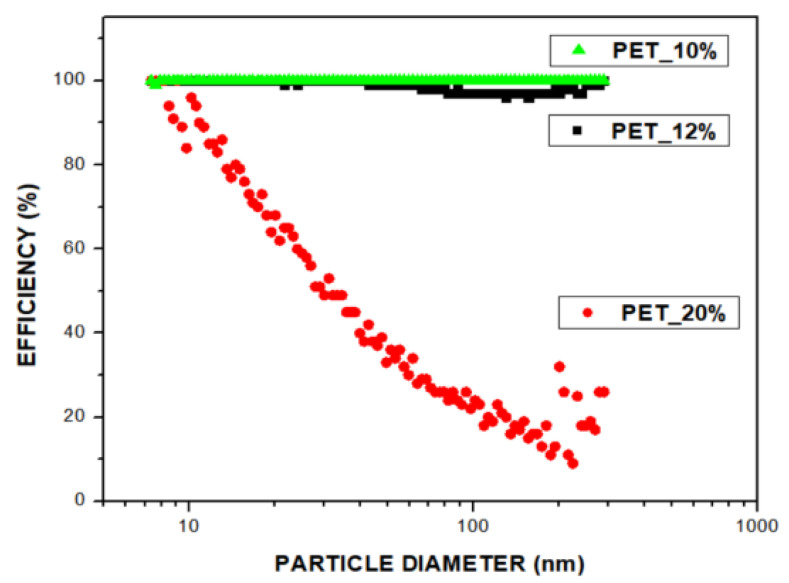
Efficiency curves for samples with filtration velocity equal to 4.8 cm s^−1^ and particle diameter distribution ranging from 7 to 300 nm.

Figure 4 shows the grade efficiency of samples PET20%, PET12%, and PET10% for a filtration velocity equal to 4.8 cm s^−1^.

It is possible to observe by analyzing the curves in Figure 4 that there is a drop in the efficiency of the PET20% and PET12% samples in relation to the PET10% sample in the particle diameter range of 100 to 250 nm. As shown in Table 4, in this interval, the efficiency of the PET20% sample drops to 18% and the efficiency of the PET12% sample drops to 96%, characterizing a region of minimum efficiency. This drop in the curve corresponds to the most penetrating particle size (MPPS) and, for particles whose diameter is smaller than the MPPS, there is a predominance of the diffusion mechanism, and for particles larger than the MPPS, interception predominates [47,48]. For the samples in the range between 100 and 250 nm, due to the greater penetration of particles in the filter, the filtration becomes less efficient, considering that the particles are too large for an effective diffusion effect, but are still too small for a significant impact of interception [21,29,30].

**Figure 5:**
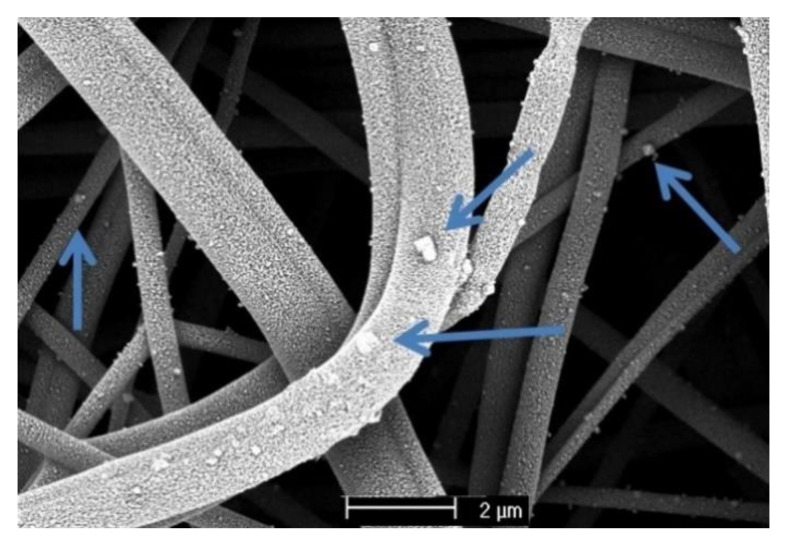
SEM images of the filter media PET_10% sample after filtration.

Figure 5 shows a microscopy image of the PET_10% sample after filtration tests, where it is possible to see NaCl nanoparticles stuck to the fiber surface. In the images, the fibers appear to be textured, but this visual effect is caused by the gold deposited to perform the SEM analysis.

The preparation of filter media by the electrospinning technique involves a series of parameters that require high control. Since an isolated characteristic of a filter media does not guarantee high efficiency, it is necessary that the combination of all factors is favorable, ideally a perfect arrangement between permeability, porosity, thickness, fiber diameter, and pressure drop. Considering that balance between conditions, the quality factor (Qf) is often used to evaluate the overall performance of filter media, because it combines the effect of collection efficiency (considering the global collection efficiency for particles whose diameter ranges from 7 to 300 nm) and pressure drop. The values obtained for each sample are shown in Table 5. Typically, the higher the Qf, the better the filtration ability of the membrane, but the Qf is influenced by the face velocity (Huang et al., 2017). Generally, a higher face velocity will lead to a lower Qf value. Several researchers have analyzed the quality factor effect in nanofiber filters [9,11,29,46].

The samples presented similar quality factors, with the best quality factor being associated with sample PET10%_0.55. Interestingly, although sample PET12% has better filtration efficiency than sample PET20%, its quality factor is lower. This fact may be associated with the high pressure drop presented in the filtration tests, as the Qf evaluates the efficiency versus pressure drop ratio. The data obtained can be compared with some filter media reported in the literature. Leung and co-workers (2010) reported a satisfactory quality factor close to 0.02 Pa^−1^ when the velocity was 5 cm s^−1^ [11], while Busher and contributors (2013) presented a theoretical analysis of the expected filtration performance and obtained membranes with 1 µm mean fiber diameter whose quality factor was less than 0.01 Pa^−1^ [50]. Bortolassi and co-workers also obtained similar quality factor media (0.04 Pa^−1^ and 0.06 Pa^−1^) for low filtration velocity (5 cm s^−1^) for PAN nanofibers [29]. Although a direct comparison among these results cannot be performed due to different experimental conditions, the Qf of the filter media prepared in this work is higher than the results reported in the literature.

Finally, the best result was obtained from the PET10%_0.55 sample, which presented an average fiber diameter of 0.66 μm, a system pressure drop of 19.4 Pa, a collection efficiency of 99%, and a quality factor of 0.35 Pa^−1^. Despite that, it is interesting to note that the results obtained for microfibers, such as for the PET12% sample, are in agreement with those found in the literature, where the pressure drop values for HEPA filters are close to 269 Pa [28]. Thus, although the PET12% filter media does not consist of nanofibers, it presented favorable characteristics for application in air filtration operations, such as high collection efficiency (99%) and relatively low-pressure drop (212.5 Pa). Thus, the manufacturing of highly efficient micro- and nanofiber filter media by electrospinning from recycled material to be applied in air filtration processes has been shown to be fully viable.

## 4. Conclusions

The objective of this study has been reached. Filter media were fabricated by electrospinning recycled PET bottles. The mean diameter was reduced from 3.25 μm to 0.65 μm in accordance with the decrease in PET concentration from 20 to 10% by weight. In addition to that, the study of the influence of the operational parameters was a key factor for obtaining different filter media, where the variation of solution concentration was the factor that most affected the diameter of the fibers produced. A decrease in the needle diameter led to a reduction in the average diameter of the nanofibers formed. The electrospinning processing time and rotation speed influenced the morphology of the fibers, mainly in the thickness and microstructural parameters of filter media. Thus, by investigating the effect of morphological characteristics on filtration performance, it was possible to obtain a filter that showed a global collection of nanoparticles of approximately 99%, a low-pressure drop of 19.4 Pa, a quality factor of 0.35 Pa^−1^, and great mechanical properties. Thus, it was possible to eliminate the microporous substrate which is used as a support for micro- and nanofibers. Therefore, it can be concluded that electrospun polymer membranes obtained from recycled materials are a promising material for applications in air filtration. It was also possible to verify that it is possible to obtain more efficient filter media with a lower pressure drop by modifying the variables, such as: concentration of the polymer, needle diameter, rotation speed, and electrospinning processing time, among others.

## Figures and Tables

**Table 1:** Operating parameters for electrospinning.

Samples	Polymer Concentration (% p/p)	Needle Diameter (mm)	Total Time of Collection (h)	Collector Speed (rpm)
PET20%	20	0.7	6	357
PET12%	12	0.7	6	357
PET10%	10	0.3	3	357
PET10%_0.3	10	0.3	3	177
PET10%_0.55	10	0.55	3	177

**Table 2:** Morphological characteristics of the fibers.

Samples	Polymer Concentration (% p/p)	Mean Fiber Diameter (μm)	Thickness (μm)	Tensile Strength (MPa)
PET20%	20	3.25	392.50	3.2
PET12%	12	1.29	342.73	3.5
PET10%	10	0.67	220.79	4.5
PET10%_0.3	10	0.65	198.01	-
PET10%_0.55	10	0.66	186.00	-

**Table 3:** Minimum and maximum pore size and porosity for each sample.

Samples	Minimum Pore Area (µm^2^)	Maximum Pore Area (µm^2^)	Porosity (Ergun Equation) (%)
PET20%	1.33	829.51	95.0 ± 0.04
PET12%	0.07	135.06	92.4 ± 0.05
PET10%	0.08	27.28	96.7 ± 0.05

**Table 4:** Characteristics of performance filtration.

Samples	Permeability Constant K_1_ (m^2^)	Pressure Drop (Pa) (*v* = 4.8 cm/s)	Global Collection Efficiency (%) (Particle Diameter 7 to 300 nm)	Fractional Collection Efficiency (%) (Particle Diameter 100 to 250 nm)
PET20%	2.2 × 10^−7^	13.5	41	18
PET12%	1.07 × 10^−8^	212.5	99	96
PET10%	3.6 × 10^−8^	76.1	99	100
PET_10%_0.3	2.6 × 10^−8^	69.8	100	100
PET_10%_0.55	6.3 × 10^−8^	19.4	99	99.4

**Table 5:** Quality factor of different filters.

Samples	Quality Factor (Pa^−1^)
PET20%	0.04
PET12%	0.02
PET10%	0.06
PET10%_0.3	0.12
PET10%_0.55	0.35
